# Experimental observation of roton-like dispersion relations in metamaterials

**DOI:** 10.1126/sciadv.abm2189

**Published:** 2021-12-01

**Authors:** Julio Andrés Iglesias Martínez, Michael Fidelis Groß, Yi Chen, Tobias Frenzel, Vincent Laude, Muamer Kadic, Martin Wegener

**Affiliations:** 1 Institut FEMTO-ST, UMR 6174, CNRS, Université de Bourgogne Franche-Comté, Besançon, 25030, France.; 2 Institute of Applied Physics, Karlsruhe Institute of Technology (KIT), Karlsruhe 76128, Germany.; 3 Institute of Nanotechnology, Karlsruhe Institute of Technology (KIT), Karlsruhe 76128, Germany.

## Abstract

Previously, rotons were observed in correlated quantum systems at low temperatures, including superfluid helium and Bose-Einstein condensates. Here, following a recent theoretical proposal, we report the direct experimental observation of roton-like dispersion relations in two different three-dimensional metamaterials under ambient conditions. One experiment uses transverse elastic waves in microscale metamaterials at ultrasound frequencies. The other experiment uses longitudinal air-pressure waves in macroscopic channel–based metamaterials at audible frequencies. In both experiments, we identify the roton-like minimum in the dispersion relation that is associated to a triplet of waves at a given frequency. Our work shows that designed interactions in metamaterials beyond the nearest neighbors open unprecedented experimental opportunities to tailor the lowest dispersion branch—while most previous metamaterial studies have concentrated on shaping higher dispersion branches.

## Teaser

Artificial materials support elastic or acoustic waves whose travel direction continuously changes from backward to forward.

## INTRODUCTION

In 1962, the Nobel Prize in Physics was awarded to L. Landau “for his pioneering theories for condensed matter, especially liquid helium.” Among other achievements, he suggested an unusual kind of dispersion relation for acoustical waves in superfluid ^4^He at low temperatures, commonly referred to as the roton (*1*). On the basis of his (*1*) and R. Feynman’s (*2*, *3*) subsequent work, rotons were observed in inelastic neutron scattering experiments in 1961 (*4*). Briefly, for the roton dispersion relation, energy and momentum of the wave are proportional to each other for small momenta. For larger momenta, a roton minimum of energy versus momentum occurs (*5*). Detailed theoretical (*6*–*10*) and experimental (*11*–*13*) investigations of this highly unusual dispersion relation in liquid ^4^He remain subject of research until today (*14*). Rotons and roton-like dispersion relations, respectively, have also been investigated experimentally in other correlated quantum systems at low temperatures, such as quasi–two-dimensional thin films of ^3^He (*15*), phonons along the (111) direction in solid helium (*16*), quantum Hall effect stripes in semiconductors (*17*–*19*), and Bose-Einstein condensates of atoms (*20*–*25*).

In 2020 (*26*) and 2021 (*27*, *28*), respectively, two papers showed by theoretical calculations that roton-like dispersion relations may also occur in designed crystals or periodic metamaterials (*29*, *30*). Here, quantum effects and correlations would play no role, and low temperatures, which often hinder applications, would not be needed. The first paper (*26*) builds on micropolar continuum elasticity theory (*31*–*33*). In this context, chirality based on broken centrosymmetry is a necessary condition for coupling to micro-rotations and hence for obtaining rotons (*26*). The second paper (*27*) suggests achiral and chiral three-dimensional (3D) periodic micro- and macrostructures. Here, the mechanism for rotons is based on tailored third-nearest-neighbor interactions in addition to the usual nearest-neighbor interactions. For pronounced roton behavior, the effective strengths of the two interactions must be comparable.

Here, we follow the specific achiral structure blueprints of the second approach (*27*) and manufacture corresponding 3D polymer–based metamaterials by 3D additive manufacturing. For each unit cell, we measure the nanometric displacement vectors for the 3D polymer–based microstructures and the scalar air pressure modulation for the macroscopic airborne-sound samples, respectively. The unusual acoustical-phonon dispersion relations obtained by Fourier transformation agree well with calculated roton band structures and with numerical finite-element calculations for the finite-size samples. We show that the roton minimum is captured qualitatively by a generalized wave equation from an analytical higher-order gradient effective medium theory.

More broadly speaking, throughout the past two decades, a large number of scientific studies have worked on shaping wave propagation in metamaterials for higher phonon bands, including phononic bandgaps, stop bands, topological bandgaps, local resonances, Dirac points, Weyl points, etc. Strangely, our ability to shape wave propagation for the lowest phonon band has fallen behind. Tailoring interactions beyond the nearest neighbors opens a systematic route toward obtaining a large variety of behaviors for the lowest band. Here, we show that these possibilities are accessible experimentally and can be tailored toward wanted frequency ranges. Roton-like dispersion relations serve as an early example.

## RESULTS

### Metamaterial blueprints and effective medium description

**Fig. 1.**
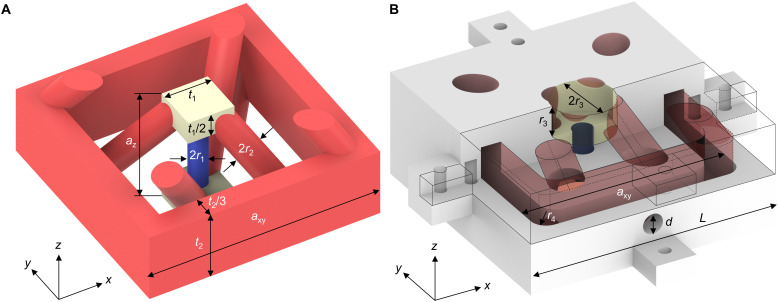
Two blueprints for roton metamaterials. (**A**) 3D microstructure unit cell of a metamaterial beam (*26*) supporting transverse-like rotons for elastic wave propagation along the *z*-direction. The blue and red cylinders are responsible for the nearest-neighbor interactions and third-nearest-neighbor interactions, respectively, between the yellow masses. Different colors are for illustration only; the entire structure is made from a single polymer material. The geometrical parameters are indicated. (**B**) Unit cell of the channel-based metamaterial beam supporting rotons for airborne longitudinal pressure waves along the *z*-direction in the channel system. This unit cell is roughly complementary to the unit cell in (A) and is composed of a bottom piece and an upper piece, whose front half is intentionally removed to show the inner compartment (yellow). The cylindrical channels for air pressure propagation are rendered semitransparent in red and blue, respectively, in analogy to (A). Here, the masses in (A) correspond to cylindrical compartments. A microphone is installed in the through hole with diameter *d* on the front wall of the lower piece. The other holes are for alignment and assembly. Only geometrical parameters different from those in (A) are given in (B).

The blueprints for the two achiral 3D tetragonal-symmetry metamaterial structures suggested theoretically in (*27*) and investigated experimentally here are illustrated in Fig. 1. For the elastic structure shown in Fig. 1A, transverse-like and longitudinal-like waves propagate in the constituent elastic material and not in the voids within. Only the transverse-like waves will show the roton behavior. For the structure in Fig. 1B, only longitudinal airborne pressure waves propagate in the voids within the material, and the material itself ideally merely provides rigid (Neumann) boundaries to the air flow in the channel system. In other words, Fig. 1B is roughly the geometrical complement of Fig. 1A. In this work, we have made minor readjustments with respect to perfect complementarity to ease the manufacturing. The waves in the structure in Fig. 1B are closer to rotons in superfluid helium than the ones in Fig. 1A in the sense that both are longitudinal waves. Both architectures can be scaled to different absolute lattice constants *a_xy_* = 2*a_z_*. We choose to realize the architecture in Fig. 1A in microscopic form with *a_z_* = 100 μm and that in Fig. 1B in macroscopic form with *a_z_* = 5 cm. Correspondingly, the two architectures operate in rather different frequency ranges: (i) at ultrasound and (ii) at audible frequencies. Both metamaterial beam samples considered have *N_z_* = 50 metamaterial unit cells along the propagation direction (*z* axis) and a finite cross section of (i) *N_x_* × *N_y_* = 2 × 2 and (ii) *N_x_* × *N_y_* = 1 × 1 unit cells in the perpendicular *xy* plane, respectively.

The mechanism leading to roton dispersion relations is the same for both metamaterials (*27*). The small cubic volumes (light yellow) act as “atoms,” i.e., as masses and small gas reservoirs, respectively. The short (blue) cylindrical rods and channels, respectively, mediate the nearest-neighbor interaction (*N* = 1) between the atoms. All other beams and channels (red), respectively, serve to mediate the third-nearest-neighbor interaction (*N* = 3). Intuitively, the length and cross section of the cylindrical elements effectively determine the strength of the corresponding interaction.

In (*27*), we have argued that the interplay of *N* = 1 and *N* = 3 contributions leads to a phonon mode hybridization and thereby to extraordinary Bragg reflections that give rise to the occurrence of the roton minimum within the first Brillouin zone of the metamaterial crystal. While this statement is valid, it is not necessary to invoke Bragg reflections to understand or reproduce the roton minimum. To appreciate this point, we make the transition to an effective medium description. As usual, the effects of Bragg reflections do not occur in effective medium continuum theory, for which one considers the limit *a_z_* → 0. For simplicity and corresponding to our experiments, we consider only waves propagating along the *z*-direction with wave number *k_z_* for both, airborne pressure waves with the air-pressure modulation $\tilde{P}$, and elastic waves with the transverse displacement *u*. The wave amplitude, *A*, shall stand for either $\tilde{P}$ or *u*. For an infinitely extended periodic lattice, we have the equation of motion for the amplitude *A_n_* = *A_n_*(*t*) at the integer lattice sites *n* = *z*/*a_z_* along the *z*-direction

$$\frac{d^{2}A_{n}}{dt^{2}}=C_{1}(A_{n+1}-2A_{n}+A_{n-1})+C_{3}(A_{n+3}-2A_{n}+A_{n-3})$$ (1)

with the nearest-neighbor coupling coefficient *C*_1_ > 0 and the third-nearest-neighbor coupling coefficient *C*_3_ ≥ 0 (cf. Supplementary Text). In the continuum limit, this equation of motion turns into the following generalized wave equation for the amplitude field *A* = *A*(*z*, *t*)

$$\frac{\partial^{2}A}{\partial t^{2}}=c_{2}\frac{\partial^{2}A}{\partial z^{2}}+c_{4}\frac{\partial^{4}A}{\partial z^{4}}+c_{6}\frac{\partial^{6}A}{\partial z^{6}}$$ (2)

with the three coefficients $c_{2}=C_{1}a_{z}^{2}+9C_{3}a_{z}^{2}>0$, $c_{4}=6C_{3}a_{z}^{4}\geq 0$, and $c_{6}=C_{3}a_{z}^{6}\geq 0$. The first two terms form an ordinary wave equation, whereas the fourth-order and sixth-order spatial derivatives with respect to *z* on the right-hand side, which are proportional to *C*_3_, specifically originate from the third-nearest-neighbor interactions. Using a plane-wave ansatz *A*(*z*, *t*) = *B* cos (*k_z_z* − ω*t*), with constant prefactor *B*, we obtain the dispersion relation for the wave’s angular frequency ω versus wave number *k_z_*

$$\omega (k_{z})=\omega (-k_{z})=\sqrt{c_{2}k_{z}^{2}-c_{4}k_{z}^{4}+c_{6}k_{z}^{6}}$$ (3)

For the above coefficients *c*_2_, *c*_4_, and *c*_6_, the root on the right-hand side does not become negative, and hence, the angular frequency ω is real valued. For small *k_z_*, the dispersion relation approximately starts as $\omega (k_{z})=\sqrt{c_{2}}\;k_{z}$, with phase velocity $\sqrt{c_{2}}$. For sufficiently large ratio *C*_3_/*C*_1_, with increasing *k_z_*, ω(*k_z_*) exhibits a maximum due to the negative fourth-order term, followed by the roton minimum due to the positive sixth-order term at yet larger *k_z_*. Below, we will plot this approximate analytical effective medium roton dispersion relation together with the more complete numerically calculated and the measured roton dispersion relations. In addition to the region of negative slope, dω/d*k_z_* < 0, which results in backward waves, it contains an angular-frequency region in which one gets three solutions for the wave number *k_z_* at a given angular frequency ω. In the limit of *k_z_* → π/*a_z_*, the higher-order gradient effective medium roton dispersion relation has the wrong asymptotics as it does not include the effects of Bragg reflections.

### The two experiments

The microscale-sample experiments have been performed at Karlsruhe, the macroscale-sample experiments at Besançon. While many details are different, conceptually, the two experiments are closely similar: We launch a wave at one end of the finite-length metamaterial beam by a monochromatic excitation source, the frequency *f* = ω/(2π) of which is varied in small frequency steps Δ*f*. We detect the resulting wave amplitude *A_n_*(*t*) versus real time *t* in each of the *n* = 1,2, …, *N_z_* = 50 metamaterial unit cells at each frequency. We extract the complex-valued Fourier components, ${\tilde{A}}_{n}(\omega )$, at frequency ω. At each frequency, we normalize the data to unity power density, i.e., $\sum_{n=1}^{50}{|{\tilde{A}}_{n}(\omega )|}^{2}=1$. In this manner, we eliminate the frequency response of the excitation process. Fourier transformation from real space to wave number leads to *N_z_* = 50 points spaced by Δ*k_z_* = 2π/(*N_z_a_z_*) within the metamaterial first Brillouin zone, hence, to 26 wave number values in the interval *k_z_* ∈ [0, π/*a_z_*]. Before the Fourier transformation, we multiply a Hann window (*34*) onto the data to suppress possible artifacts from the two sample ends at *n* = 1 and *n* = 50, respectively. In the graphical representations below, we omit the negative wave numbers due to the symmetry ω(*k_z_*) = ω(−*k_z_*). The other end of the beam is closed for the channel-based pressure wave system and left open for the elastic system, such that in both cases 100% of the wave is reflected at that end. In the presence of finite wave damping, the resulting waves are therefore a mixture of standing waves and propagating waves along the metamaterial beam axis. At the launching end, the surface termination decides how well the excitation couples to the different modes at a given angular frequency, thus it determines the weight of the mode in the band structures to be shown below. We have optimized the surface terminations of the two 3D structures for roughly equal weights based on numerical finite-element calculations to avoid a tedious experimental trial-and-error procedure. In the higher-order gradient effective medium description outlined in the previous section, the effect of the surface termination, which is not expected for ordinary acoustical phonons, enters via the fourth- and sixth-order spatial derivatives, which must be matched at an interface in addition to the first- and second-order spatial derivatives.

**Fig. 2.**
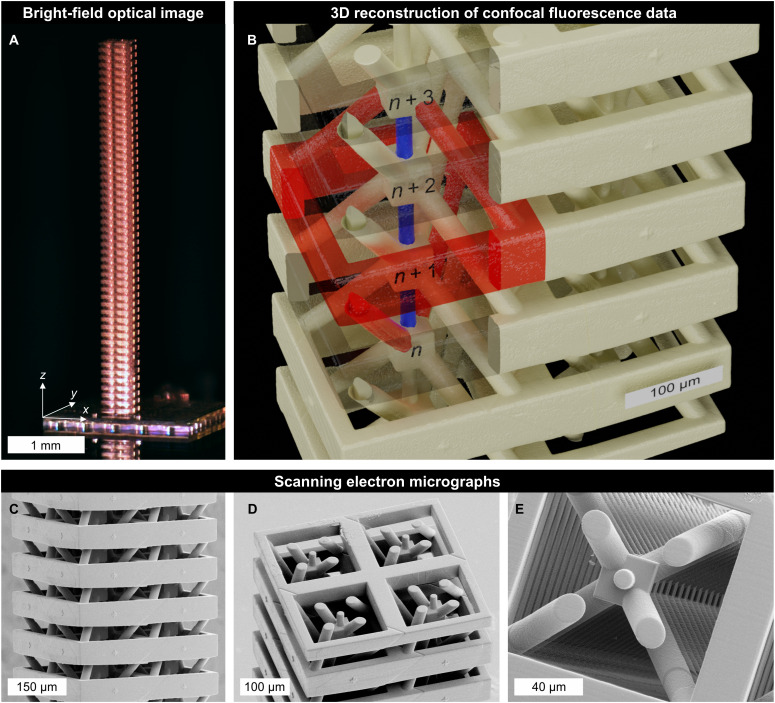
Roton metamaterial microstructures for elastic waves. The shown polymer samples, which have been manufactured in one piece by multiphoton 3D laser microprinting, follow the blueprint shown in Fig. 1A. (**A**) Overview of a sample imaged with a wide-field microscope. Photo credit: M. F. Groß (Karlsruhe Institute of Technology) and T. Frenzel (Karlsruhe Institute of Technology). (**B**) 3D iso-intensity surface acquired with a confocal fluorescence optical microscope (LSM 800, Zeiss) using the autofluorescence of the polymer. Scale bars and labels added in postprocessing using blender. Parts of the unit cell frames are made to appear transparent to reveal the interior. The third-nearest-neighbor coupling is colored in red, the nearest-neighbor coupling is colored in blue. (**C** to **E**) Scanning electron micrographs of (C) the unit cell frames, (D) the uppermost layers, and (E) a view along the center axis of one column of unit cells.

**Fig. 3.**
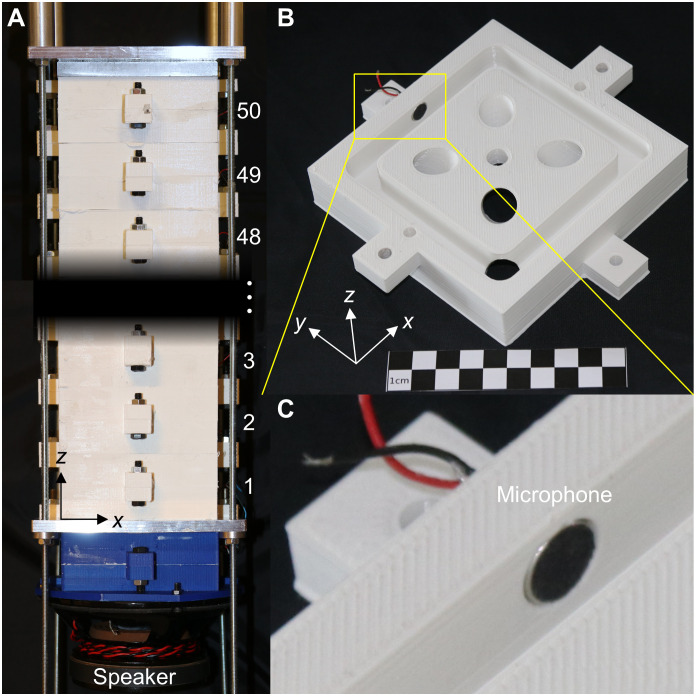
Roton metamaterial sample for airborne sound. (**A**) The 3D-printed polymer sample follows the blueprint shown in Fig. 1B. It has been assembled from 100 individual pieces, 2 for each of the *N_z_* = 50 unit cells. The metamaterial sample has a length of 2 m along the *z*-direction. Therefore, only the bottom part and the top part are shown here. (**B**) One of the two pieces for one unit cell. (**C**) Zoom-in view of the highlighted rectangle region in (B) showing an installed microphone on the side wall. Photo credit: J. A. Iglesias Martínez (Université de Bourgogne Franche-Comté).

Figure 2 describes and illustrates the microscale samples, and Fig. 3 describes the macroscale samples. For the manufacturing of the polymer samples shown in Fig. 2, we have used a standard commercial 3D laser nanoprinter (Photonics Professional GT, Nanoscribe) and a commercial photoresist (IP-S, Nanoscribe). Details of the manufacturing process, in which one must cope with a large number of strongly overhanging parts, are given in Methods and Materials. To ease the tracking of the transverse and longitudinal components of the displacement vectors, we have added a set of cross-shaped markers (*35*) onto the frame of each unit cell along the *z*-direction. The markers represent a negligible perturbation with respect to the wave propagation. The macroscopic samples in Fig. 3 have been subdivided into 100 pieces, 2 pieces for each of the *N_z_* = 50 unit cells. The individual pieces have been manufactured commercially by 3D printing (fused deposition modeling with polylactic acid). Each unit cell contains a cylindrical hole with a diameter of 9.8 mm in the frame to insert a microphone (Kepo, KPCM-94H65L-40DB-1689). The microphones have been glued into the holes and the 100 pieces have been glued and tightly screwed together to achieve an air channel system that is sealed with respect to the outside.

Let us briefly give a few more specifics for the two different experiments. For the measurements on the samples in Fig. 2, we excite the samples by a piezoelectric actuator (PL055.31 PICMA, Physik Instrumente). The samples are imaged from the side by a home-built scanning confocal optical microscope comprising a continuous-wave laser (LCX-532S-200, Oxxius SA) operating at 532 nm wavelength, a microscope objective lens (50× CFI60 TU Plan Epi ELWD, Nikon), and a pinhole in an intermediate imaging plane for moderate *z*-sectioning (cf. Supplementary Text and fig. S1). The nanometric transverse and longitudinal components of the displacement vectors of all unit cells at the cross marker positions are obtained by means of optical image digital cross-correlation analysis (*35*, *36*) on a computer (cf. Supplementary Text and figs. S2 and S3). For the measurements on the sample in Fig. 3, we excite the sample by a loudspeaker (Fane, Sovereign 6-100). We record the local air-pressure modulation by a microphone (Kepo, KPCM-94H65L-40DB-1689) in each of the *N_z_* = 50 unit cells. We feed the electrical analog signals into the sound card of a computer, where the data are further processed. For further details, see Materials and Methods.

**Fig. 4.**
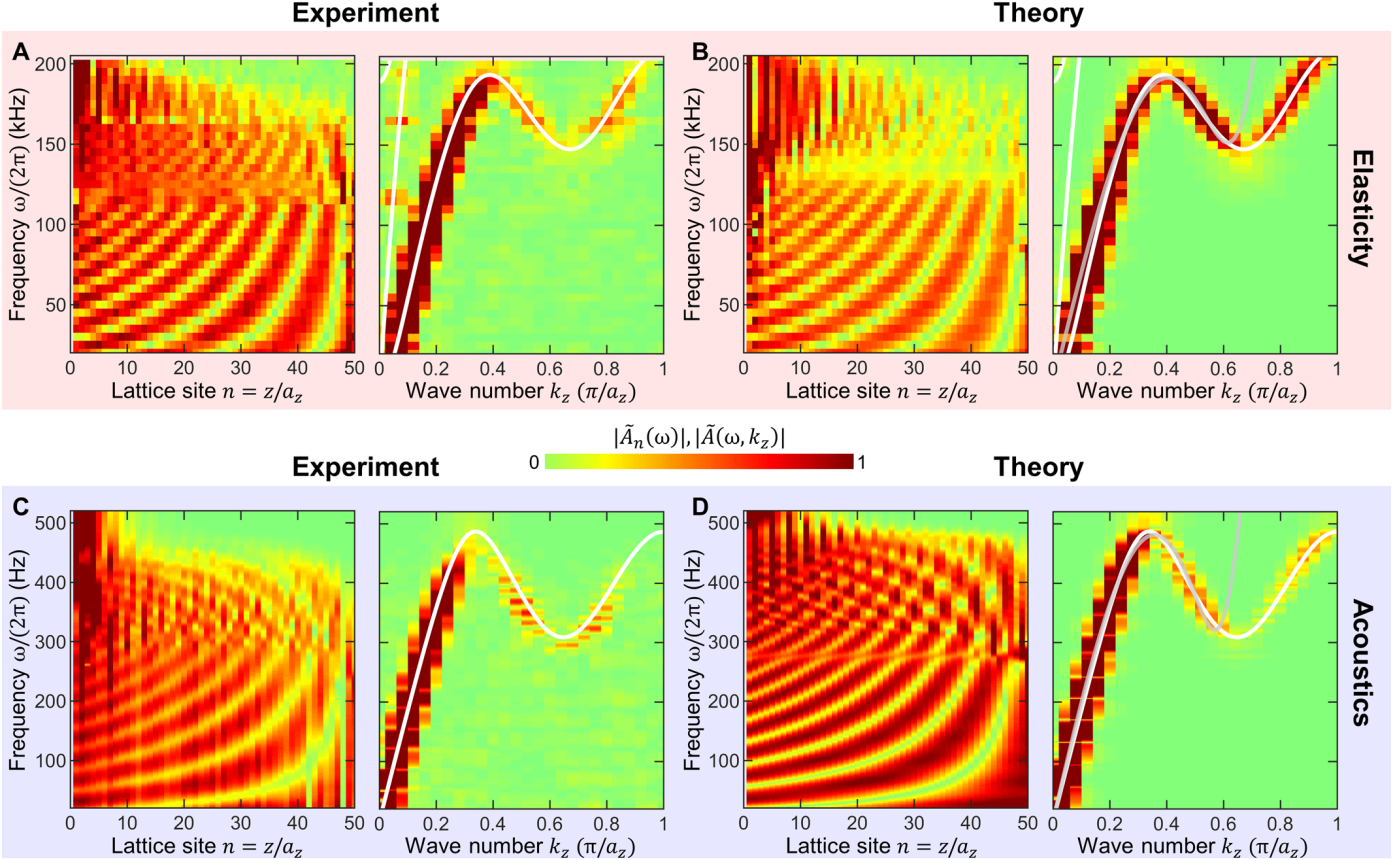
Measured and calculated roton dispersions. (**A**) Measured raw data (left) for the sample in Fig. 2 versus position and frequency and derived roton band structure (right). (**B**) Corresponding numerically calculated behavior for the same finite sample length and including damping. (**C**) As in (A) but for the sample in Fig. 3. (**D**) Numerically calculated behavior corresponding to the measurements in (C). The white solid curves are the calculated roton band structures for a lossless metamaterial beam that is infinitely extended along the *z*-direction. For the elastic metamaterial, we use the geometrical parameters: *a_xy_* = 200 μm, *a_z_* = 100 μm, 2*r*_1_ = 16.8 μm, 2*r*_2_ = 25.2 μm, and *t*_2_ = 60 μm. For the airborne metamaterial, we use the geometrical parameters: *a_xy_* = 100 mm, *a_z_* = 50 mm, 2*r*_1_ = 10 mm, 2*r*_2_ = 16 mm, 2*r*_3_ = 30 mm, *t*_2_ = 30 mm, *d* = 9.8 mm, *r*_4_ = 7.5 mm, and *L* = 120 mm. The gray curves in (B) and (D) correspond to the approximate analytical dispersion relations of the higher-order gradient effective medium model with parameters *c*_2_, *c*_4_, and *c*_6_ fitted to the interval *k_z_* ∈ [0,0.6 × π/*a_z_*].

Figure 4 summarizes resulting exemplary measured raw data for the two experiments and the derived roton band structures within the first Brillouin zone with ∣*k_z_*∣ ≤ π/*a_z_*, represented on false color scales alongside corresponding theoretical calculations for the finite-length (*N_z_* = 50) metamaterial beams. In these calculations, damping effects are included phenomenologically (see Materials and Methods). The additional white solid curves are the result of band structure calculations for fictitious lossless metamaterial beams that are infinitely periodic along the *z*-direction. Here, we use Floquet-Bloch periodic boundary conditions along the *z*-direction and traction-free boundary conditions along the *x*- and the *y*-direction. We use identical geometrical and material parameters as above. For clarity, for the elastic case, we do not depict the twist band in Fig. 4 because the piezoelectric excitation does not couple to it. The gray solid curves are the approximate analytical roton dispersion relations of the higher-order gradient effective medium model introduced above.

In Fig. 4, we present two metamaterial experiments that provide evidence for roton dispersion relations under ambient conditions. The experimental data for the ultrasound transverse-like elastic waves (cf. Fig. 2) shown in Fig. 4A and for the audible sound airborne longitudinal pressure waves (cf. Fig. 3) shown in Fig. 4C are in good agreement with the respective numerical calculations shown in Fig. 4 (B and D) performed for the same finite-length metamaterial beams. For the elastic waves, the dispersion relation derived from the longitudinal component of the displacement vectors (cf. fig. S4) shows an ordinary phonon dispersion relation with larger phase velocity than the transverse branch. In addition, one can see the transverse-like roton because the modes in the metamaterial beam with finite cross section have somewhat mixed transverse/longitudinal character. The finite-length sample data in Fig. 4 and fig. S4 agree well with the band structures calculated for infinitely long metamaterial beams (see white solid curves). These band structures are captured qualitatively well by the analytical dispersion relations (see gray solid curves) derived from the approximate higher-order gradient effective medium roton model introduced above.

The weight of the peaks in the false color representation of the measured roton band structures in Fig. 4 should be taken with caution, especially in the frequency region where three peaks versus *k_z_* occur at a given frequency. First, at each frequency, we have normalized the Fourier transforms to unity power density to obtain a unique and unambiguous normalization. As a result, a single peak with fixed shape versus *k_z_* will always have the same weight. Three equally strong peaks versus *k_z_* will have only one third of that weight. By definition, the normalization thereby reduces the peak heights in the roton region. Second, the experimental real-space data additionally comprise noise. This is especially true for the nanometric measurements on the elastic microstructures. The noise contains weight in the spatial Fourier power spectrum and thereby reduces the roton peak heights due to the normalization to equal power density. Third, we recall that the weight of the individual peaks at different *k_z_* can be influenced by the excitation conditions, specifically by the surface termination of the sample at the metamaterial beam end of the excitation. We have optimized the excitation conditions for roughly equal weight of the peaks. The dependence of the coupling to one of the three modes on the surface termination is a particularity of rotons. In terms of effective medium descriptions, it is a particularity of the higher-order gradient generalized wave equation.

## DISCUSSION

This particularity of the roton behavior is an opportunity for future work. At a given frequency, at the interface between an ordinary material and a material showing roton-like behavior, for a wave emerging from the ordinary material, one can choose the mode in the roton-like material one wishes to couple to via tailoring of the structure at the interface. This means that one can choose between three different wavelengths and behaviors. This aspect brings an entirely new design quality to the interface or transition region. Last, our experimental results for transverse elastic waves and longitudinal airborne pressure waves in metamaterials can likely also be transferred to other areas of physics, for example, to electromagnetism and optics.

## MATERIALS AND METHODS

### Band structure calculations

For the elastic and air-borne sound roton metamaterials, respectively, we numerically find the angular eigenfrequency ω_*i*_(**k**) for the band with band index *i* at wave vector **k** from the eigenvalue equation derived from linear elasticity theory

$$\begin{array}{c} \frac{E}{2(1+v)(1-2v)}\nabla (\nabla \cdot u_{k,i}(r))+\\ \frac{E}{2(1+v)}\nabla^{2}u_{k,i}(r)=-{\rho \omega }_{i}^{2}(k)u_{k,i}(r) \end{array}$$ (4)

and acoustic theory

$$\nabla \cdot (\nabla P_{k,i}(r))=-\frac{\omega_{i}^{2}(k)}{v_{\text{air}}^{2}}{\tilde{P}}_{k,i}(r)$$ (5)

respectively. Here, $u_{k,i}(r)$ and ${\tilde{P}}_{k,i}(r)$ represent the displacement eigenmode and pressure eigenmode for the two metamaterials, respectively. We solve these equations by using the Solid Mechanics Module and the Pressure Acoustic Module, respectively, in the commercial software Comsol Multiphysics, specifically using its multifrontal massively parallel sparse direct solver (MUMPS). Floquet-Bloch periodic boundary conditions are applied along the *z*-direction, **k** = *k_z_***e**_*z*_, of the metamaterial sample containing *N_x_* × *N_y_* = 2 × 2 and *N_x_* × *N_y_* = 1 × 1 unit cells, respectively, in its cross section. Traction-free boundary conditions are applied to all interfaces to voids (air or vacuum) for the elastic roton metamaterial. Sound rigid boundary conditions are applied to all interfaces to solids for the air-borne sound case. We choose *E* = 4.19 GPa for the Young’s modulus, ν = 0.4 for the Poisson’s ratio, and ρ = 1140 kg/m^3^ for the mass density of the constituent material of the elastic metamaterial. We choose a speed of sound in air of *v*_air_ = 340 m/s for the air-borne sound metamaterial.

### Finite-size sample calculations

The responses of the finite-size elastic roton metamaterial and the air-borne sound roton metamaterial samples are calculated in the frequency domain by using the equations

$$\begin{array}{c} \frac{E}{2(1+v)(1-2v)}\nabla (\nabla \cdot u(\omega ,r))+\\ \frac{E}{2(1+v)}\nabla^{2}u(\omega ,r)=-{\rho \omega }^{2}u(\omega ,r) \end{array}$$ (6)

and

$$\nabla \cdot (\nabla \tilde{P}(\omega ,r))=-\frac{\omega^{2}(k)}{v_{\text{air}}^{2}}\tilde{P}(\omega ,r)$$ (7)

with **u**(ω,**r**) and $\tilde{P}(\omega ,r)$ representing the displacement and the pressure modulation responses at angular frequency ω, respectively. The equations are solved by using the Solid Mechanics Module and the Pressure Acoustic Module in the commercial software Comsol Multiphysics, again with its MUMPS solver. We apply a transverse displacement field, $u(\omega ,r=0)=(u_{0},0,0)\text{cos}(\omega t)$, and a pressure modulation field, $\tilde{P}(\omega ,r=0)=P_{0}\text{cos}(\omega t)$, to the bottom end of the two samples, respectively, to model time harmonic excitation in the experiment. At the other boundaries of the samples, traction-free boundary conditions and acoustic rigid boundary conditions are applied, respectively. The material parameters are the same as in the “Band structure calculations” section except that an imaginary part of the Young’s modulus *E* and of the sound velocity *v*_air_, respectively, are used to mimic damping effects that occur in the experiments. The imaginary part is set to 5% of the real part of the Young’s modulus *E* and 2% of the real part of the sound velocity *v*_air_, respectively.

### Sample fabrication of the elastic roton metamaterial

We fabricate the microscale metamaterials using 3D laser microprinting (Professional GT, Nanoscribe GmbH). We use a 25× objective lens (numerical aperture = 0.8, Carl Zeiss), which is dipped directly into the liquid photoresist (IP-S, Nanoscribe GmbH). The laser focus is scanned using two galvanometric mirrors at a focus speed of 0.125 m/s. The mean laser power is set to 27.5 mW, measured at the entrance pupil of the microscope lens.

The underlying 3D models are created using the commercial software package COMSOL Multiphysics (COMSOL Inc.). Subsequently, these are transcribed into machine code using the software Describe (Nanoscribe GmbH). We choose a hatching distance of 300 nm, a slicing distance of 700 nm, and a stitching distance of 200 μm.

Since pending and overhanging structures cannot be fabricated easily, the unit cell is split into several parts that are printed consecutively. To ensure a single connected sample during the whole printing process, the writing direction is switched multiple times between +*z* and −*z*. Furthermore, we add support structures and scaffoldings, which become part of the structure in a later printing step and therefore do not need to be removed. More details can be found in the original GWL-files that are published in (https://doi.org/10.35097/488).

Furthermore, a bottom plate is added to facilitate handling of the samples and to ensure proper contact to the piezoelectric element when being glued. This bottom plate is printed using a focus speed of 0.140 m/s, a laser power of 50 mW, a hatching distance of 0.5 μm, and a slicing distance of 1.5 μm.

After exposure, the excess photoresist is removed in a beaker of MR-Dev 500 and acetone for 20 min each. This step is followed by critical point drying (Leica EM CPD300, using CO_2_).

### Elastic experiment setup

The elastic metamaterial sample is glued onto the side of an aluminum cuboid, which is fixed to a piezoelectric actuator such that the main axis of the actuator lies in the transverse plane of the metamaterial sample. Since the metamaterial sample exceeds the optical field of view of the confocal optical microscope setup, the actuator is mounted on an *xyz* translation stage using piezo inertia drives for sample manipulation. This assembly is positioned in the focal plane of the microscope objective lens (cf. fig. S1) to enable a side view of the metamaterial sample. The back-reflected light is measured with an avalanche photodiode module that outputs a photovoltage proportional to the incident light power.

### Elastic single-frequency excitation measurement

The metamaterial sample is excited by driving the piezoelectric actuator with an amplified sinusoidal voltage. The data acquisition unit of the confocal optical microscope is synchronized to this excitation signal. This synchronization is crucial for image generation and for subsequently obtaining the phase information of the displacement trajectory at each sample position. Data acquisition consists of scanning a spatial region of interest (ROI) on the excited sample. The ROI size is chosen to span 60 × 60 pixels over a rectangular sample area of 30 μm × 30 μm. At each pixel, a time series of the photovoltage is acquired before the next pixel is measured. A total of 51 ROIs are investigated for each sample. The zeroth ROI is located on the base plate of the sample providing quantitative information about phase and amplitude of the sample excitation. The remaining 50 ROIs are placed on the cross-shaped markers on the outer frame of each metamaterial layer.

The frequency resolution of the excitation is set to Δ*f* = 5 kHz. The excitation frequency is kept constant until all ROIs have been measured for that frequency. Between switching of the excitation frequency and starting a new time series, the data acquisition is halted for about 200 ms. This waiting time acts as a buffer for the sample to respond to the new excitation signal. Images for the digital image cross-correlation analysis are generated by combining the set of time series for each frequency at every ROI, respectively, using the synchronization between the excitation signal and the data acquisition (cf. fig. S2).

### Acoustic experiment setup

Each unit cell of one acoustic metamaterial sample is divided into two identical parts, thanks to the symmetry with respect to the horizontal plane parallel to the *xy* plane. A set of 100 identical pieces is fabricated by 3D printing (3D Hubs). The two parts forming each of the 50 unit cells are joined together with the help of screws and an acrylic plastic filler (Mastic, Axton) to ensure sealing with respect to air. In each unit cell, a microphone is positioned inside a hole on the side of a half unit cell. The hole in the other half is closed with hot glue. A loudspeaker (diameter 165 mm) is fixed to an extra unit cell printed with a circular face (cf. fig. S5). The extra unit cell is added at the entrance of the metamaterial sample to couple sound into the channel-based metamaterial sample. The other end of the metamaterial beam is terminated by a metal plate to obtain boundary conditions comparable to the elastic case. The entire metamaterial structure is compressed by threaded rods to ensure mechanical stability (cf. fig. S6).

### Acoustic single-frequency excitation measurement

A sinusoidal signal at a given frequency is produced by a computer, subsequently amplified, and sent to the loudspeaker. Using the sound card of the computer, the signals received by two microphones are recorded simultaneously, at the *N*th unit cell and at the entrance unit cell where the excitation is fed into. The latter signal is used as a reference for phase and amplitude measurements. The sinusoidal signal duration is set to 1 s, resulting in a 1 Hz frequency resolution. The measurement is performed for all frequencies of interest and for the *N_z_* = 50 U cells, including a 1 s silence between each recording to ensure that a silent steady state is recovered. To further validate the single-frequency excitation measurements, additional experiments with white noise excitation have also been performed. The obtained first band agrees well with that obtained by single-frequency excitation, and the higher bands agree well with corresponding theoretical calculations (cf. figs. S6 and S7).

## Supplementary Materials

### This PDF file includes:

Supplementary text Figs. S1 to S7 Table S1 References
