# Supplementary material for: Experimental observation of roton-like dispersion relations in metamaterials
Source: Sci Adv. 2021 Dec 1;7(49):eabm2189. doi: 10.1126/sciadv.abm2189 (PMC8635434; doi:10.1126/sciadv.abm2189)
Supplement: Supplementary file 1 — Supplementary text Figs. S1 to S7 Table S1 References [file sciadv.abm2189_sm.pdf]

Science Advances
AAAS

Supplementary Materials for

Experimental observation of roton-like dispersion relations in metamaterials

Julio Andrés Iglesias Martínez, Michael Fidelis Groß, Yi Chen*, Tobias Frenzel, Vincent Laude, Muamer Kadic, Martin Wegener*

*Corresponding author. Email: yi.chen@partner.kit.edu (Y.C.); martin.wegener@kit.edu (M.W.)

Published 1 December 2021, *Sci. Adv.* **7**, eabm2189 (2021)
DOI: 10.1126/sciadv.abm2189

This PDF file includes:

Supplementary text
Figs. S1 to S7
Table S1
References

Supplementary Text

Higher-order-gradient effective-medium approximation

In the main paper, we have considered the equation of motion for the wave amplitude A_n at integer lattice site n

$$\frac{\mathrm{d}^2 A_n}{\mathrm{d}t^2} = C_1(A_{n+1} - 2A_n + A_{n-1}) + C_3(A_{n+3} - 2A_n + A_{n-3}). \quad \text{(S1)}$$

For the case of elastic waves, where $A_n = u_n$ stands for the transverse displacement u_n of a mass m, we start from the mass-and-spring toy model discussed in (*27*). It is immediately obvious that the coupling coefficients are given by $C_1 = K_1/m$ and $C_3 = K_3/m$, with the Hooke's spring constants K_1 and K_3.

For the case of the channel- or tube-based metamaterial for airborne sound, the wave amplitude is given by the pressure modulation, i.e., $A_n = \tilde{P}_n$. The masses in the elastic case are replaced by the air compartments with volume V_c at lattice site n. The air pressure $P_n = P_\mathrm{a} + \tilde{P}_n$, with the constant ambient background pressure P_a, shall be approximated by the constant mean in that compartment. The air pressure directly translates into the number of air molecules $N_n = N_\mathrm{a} + \tilde{N}_n$ in one compartment. N_a is the number of air molecules in the compartment at fixed room temperature T, corresponding to the background pressure P_a. The ideal-gas equation reads $P_n = N_n \frac{k_\mathrm{B} T}{V_\mathrm{c}}$, or $\tilde{P}_n = \tilde{N}_n \frac{k_\mathrm{B} T}{V_\mathrm{c}}$, with the Boltzmann constant k_B. The interactions between the compartments are mediated by cylindrical tubes with inner radius R_N, hence inner cross section area πR_N^2, and length L_N. The integer N is given by $N = 1$ for the nearest-neighbor interactions and $N = 3$ for the third-nearest-neighbor interactions. From **Fig. 1(B)** and **Fig. 2**, it is clear that the assumption of cylindrical tubes is directly met in the experiments for $N = 1$. In contrast, for $N = 3$, the channel system in the experiment is more complex. Therefore, for $N = 3$, the radius R_3 and the length L_3 should be taken as approximate effective quantities. Furthermore, we approximate the air velocity in the N-th order tube connecting site n with site $n + N$, $v_n^{(N)}$, as being constant throughout that tube. The particle density, ρ_a, within all tubes is approximated as being constant, with $\rho_\mathrm{a} = P_\mathrm{a}/(k_\mathrm{B} T)$. With these definitions, the continuity equation, which describes the in-flux and out-flux of air molecules from the tubes into and out of the compartment with index n, reads

$$\frac{\mathrm{d}}{\mathrm{d}t} N_n = \frac{\mathrm{d}}{\mathrm{d}t} \tilde{N}_n = -\rho_a \pi R_1^2 \left(v_n^{(1)} - v_{n-1}^{(1)}\right) - \rho_a \pi R_3^2 \left(v_n^{(3)} - v_{n-3}^{(3)}\right). \quad \text{(S2)}$$

Taking the time derivative, we obtain

$$\frac{\mathrm{d}^2}{\mathrm{d}t^2}\widetilde{N}_n = -\rho_a\pi R_1^2\left(\frac{\mathrm{d}}{\mathrm{d}t}v_n^{(1)} - \frac{\mathrm{d}}{\mathrm{d}t}v_{n-1}^{(1)}\right) - \rho_a\pi R_3^2\left(\frac{\mathrm{d}}{\mathrm{d}t}v_n^{(3)} - \frac{\mathrm{d}}{\mathrm{d}t}v_{n-3}^{(3)}\right). \tag{S3}$$

The acceleration $\frac{\mathrm{d}}{\mathrm{d}t}v_n^{(N)}$ in the Nth-order tubes results from the net force corresponding to the pressure difference between the two ends of the tube with length L_N, i.e., from

$$\rho_a\frac{\mathrm{d}}{\mathrm{d}t}v_n^{(N)} = -\frac{\tilde{P}_{n+N}-\tilde{P}_n}{L_N}. \tag{S4}$$

Inserting (S4) into (S3) and replacing $\widetilde{N}_i = \frac{\rho_\mathrm{a}V_\mathrm{c}}{P_\mathrm{a}}\tilde{P}_i$ on the left-hand side of (S3) leads to

$$\frac{\mathrm{d}^2\tilde{P}_n}{\mathrm{d}t^2} = C_1\left(\tilde{P}_{n+1} - 2\tilde{P}_n + \tilde{P}_{n-1}\right) + C_3\left(\tilde{P}_{n+3} - 2\tilde{P}_n + \tilde{P}_{n-3}\right), \tag{S5}$$

with the coupling coefficients

$$C_N = \frac{P_\mathrm{a}}{\rho_\mathrm{a}V_\mathrm{c}}\frac{\pi R_N^2}{L_N}. \tag{S6}$$

With $A_n = \tilde{P}_n$, we get the equation of motion (S1) we have started from above.

Next, we make the transition from this equation of motion for a discrete lattice with wave amplitude A_n to an approximate generalized wave equation. As usual, the nearest-neighbor coupling term can be rewritten as a difference quotient, which turns into the spatial differential quotient in the limit of vanishingly small lattice constant, $a_z \to 0$, according to

$$C_1(A_{n+1} - 2A_n + A_{n-1}) = C_1 a_z^2\frac{(A_{n+1}-2A_n+A_{n-1})}{a_z^2} \to C_1 a_z^2\frac{\partial^2 A}{\partial z^2}. \tag{S7}$$

We proceed analogously for the third-nearest-neighbor interaction term in (S1)

$$C_3(A_{n+3} - 2A_n + A_{n-3}), \tag{S8}$$

for which we need the general form for the m-th order spatial derivative for even m. It is given by (*37*)

$$\frac{\partial^m A}{\partial z^m} \approx \frac{1}{a_z^m}\sum_{j=0}^{m}(-1)^j\frac{m!}{j!\,(m-j)!}A_{i+j-\frac{m}{2}}. \tag{S9}$$

We obtain

$$\begin{aligned} &C_3(A_{n+3} - 2A_n + A_{n-3}) \\ &= C_3 a_z^6\,\frac{A_{n+3} - 6A_{n+2} + 15A_{n+1} - 20A_n + 15\,A_{n-1} - 6A_{n-2} + A_{n-3}}{a_z^6} \\ &+ 6C_3 a_z^4\,\frac{A_{n+2} - 4A_{n+1} + 6A_n - 4A_{n-1} + A_{n-2}}{a_z^4} + 9C_3 a_z^2\,\frac{A_{n+1} - 2A_n + A_{n-1}}{a_z^2} \\ &\to C_3 a_z^6\frac{\partial^6 A}{\partial z^6} + 6C_3 a_z^4\frac{\partial^4 A}{\partial z^4} + 9C_3 a_z^2\frac{\partial^2 A}{\partial z^2}, \end{aligned} \tag{S10}$$

where the last step applies for the limit of $a_z \rightarrow 0$. Combining the terms and introducing the coefficients c_2, c_4, and c_6 leads to the generalized wave equation for the amplitude field $A(z,t)$

$$\frac{\partial^2 A}{\partial t^2} = c_2 \frac{\partial^2 A}{\partial z^2} + c_4 \frac{\partial^4 A}{\partial z^4} + c_6 \frac{\partial^6 A}{\partial z^6}, \tag{S11}$$

with $c_2 = C_1 a_z^2 + 9C_3 a_z^2 > 0$, $c_4 = 6C_3 a_z^4 \geq 0$, and $c_6 = C_3 a_z^6 \geq 0$, which we have used in the main paper. Making the plane-wave ansatz $A(z,t) = B\cos(k_z z - \omega t)$ with constant prefactor B, we immediately obtain the higher-order-gradient effective-medium roton dispersion relation

$$\omega(k_z) = \sqrt{c_2 k_z^2 - c_4 k_z^4 + c_6 k_z^6}\,. \tag{S12}$$

As discussed in the main paper, the effective-medium approximation does not include the effects of Bragg reflections. Therefore, this dispersion relation becomes a bad approximation of the true dispersion relation for wavenumbers k_z approaching the edge of the first Brillouin zone at $|k_z| = \pi/a_z$. Nevertheless, we emphasize that the approximate effective-medium dispersion relation does capture the existence of the roton minimum.

Tracking elastic waves using confocal microscopy

To determine the displacement field of elastic waves in three-dimensional metamaterials by the means of digital image cross-correlation (DIC) analysis, it is clear that the optical measurement setup must use a frame rate that is at least twice the highest oscillation frequency of the elastic waves (*38*). In the case of the microscale metamaterials investigated in this work, the frequency range of interest corresponds to ultrasound frequencies between 20 kHz and 200 kHz. Hence, a frame rate of at least around 400 kHz is required. For optical wide-field microscopy setups, this is either achievable by using expensive high-speed cameras or with the aid of stroboscopic illumination as was, for example, demonstrated by Frenzel et al. (*39*) For a confocal laser scanning optical microscope used in this work, the situation is different. In general, the frame rate is limited by the speed of the scanning instrument and not by the data acquisition unit. Usually, this results in frame rates that are even further below the acquisition rates required for imaging at ultrasound frequencies. However, photodetectors common in confocal microscopy, such as avalanche photodiodes, easily allow for a signal bandwidth in the range of 10 MHz when paired with appropriate data acquisition units. Using this configuration, it is possible to circumvent both, stroboscopic illumination and high frame rates, by acquiring time-resolved data pixelwise over the image plane. However, a necessary requirement is that the displacement field is time-periodic.

This condition is fulfilled for the experiments on the microscale metamaterial samples described in this work.
The following text and **Fig. S1** describe the setup that has been used by us for the experiments on the microscale metamaterial samples. Furthermore, a detailed description of the measurement procedure is given in **Fig. S2**. A list of components is provided in **Table S1**.

Scanning-confocal-optical-microscope setup description

The layout of the setup is sketched in **Fig. S1** and a list of components is provided in **Table S1**. The single longitudinal mode laser emits at a wavelength of $532\,\mathrm{nm}$ with a beam diameter of $0.7\,\mathrm{mm}$. A Faraday isolator next to the laser blocks back-reflections inherent to any backscattering confocal optical microscope to avoid instabilities and fluctuations of the laser power. With the first half-wave plate (R1) and a polarizing beam splitter cube (PBS), the laser power for the sample illumination is adjusted. The second half-wave plate (R2) can be adjusted to rotate the laser beam polarization if needed. To ensure an optical excitation at only $532\,\mathrm{nm}$, an optical clean-up filter is included behind the second half-wave plate. The thin-film beam splitter (TFBS) separates the illumination path from the detection path. To match the laser-beam diameter to the entrance pupil of the microscope objective lens, three $4f$ telescopes (TEL1-TEL3) are implemented before the beam reaches the galvanometer scanner. The scan lens (SC) is realized from two achromatic lenses in a Plössl configuration inspired by A. Negrean and H. D. Mansvelder (*40*). A single achromatic lens acts as a tube lens (TL) before the laser beam illuminates the aperture of the objective lens (OL). The final lens (DL) of the detection path generates the conjugate point to the focal point of the OL. Hence, the DL couples the backscattered light from the sample into a multimode fiber, which effectively serves as the pinhole of the confocal setup. Considering the focal lengths of all used lenses, the total magnification of the setup is given by $M = 24.89$. The multimode fiber is connected to the active area of an avalanche photodiode module (APD-Module), which directly outputs a voltage proportional to the incident light power. The APD-Module is linked to the data acquisition unit (DAQ) of the personal computer (PC) to measure the resulting photovoltage. The RTC6 card in the PC is responsible for controlling the galvanometer scanner and provides a trigger enable signal to the auxiliary I/O port of the DAQ. For alignment of the sample plane under investigation with the focal plane of the OL, the sample is located on a three-axis tilt stage that we adjust manually. This stage is used to compensate possible tilts of the sample that may occur when

fixing the sample to the piezoelectric actuator. Furthermore, the sample manipulation is controlled by the PC and implemented using a xyz-translation stage comprising three piezo-inertia stages. The drive signal for the mechanical sample excitation is provided by a function generator and is subsequently amplified. The same function generator also provides a synchronization signal, which is used as an external trigger for the DAQ.

Measurement procedure

The following text explains the procedure we use for obtaining the displacement field of a microscale metamaterial sample from the measured photovoltage time signal. Starting from the illustration in **Fig. S2(A)**, the sample is mechanically excited by a piezoelectric actuator at a set frequency in the range of $20\,\mathrm{kHz}$ to $200\,\mathrm{kHz}$. The required time-harmonic drive signal for the piezoelectric actuator is generated by a function generator and subsequently amplified. Once the laser beam is positioned on the first pixel of the region of interest (ROI) by the galvanometer scanner, the RTC6 card arms the DAQ via the trigger enable signal on its auxiliary I/O port. Subsequently, the acquisition of the photovoltage is triggered on the rising edge of the synchronization signal. The latter is a square-wave voltage derived from the same function generator as the drive signal. Once the time series of Z data points for one pixel is complete, the laser beam is moved to the next pixel by the galvanometer scanner and the process is repeated. The resulting set of time series for one ROI can be visualized by a cuboid, as depicted in **Fig. S2(B)**. The lateral coordinates represent spatial pixels of the ROI on the sample. Each column represents the time series of the photovoltage for an individual pixel. This depiction is meaningful because the data acquisition is synchronized to the sample excitation via the synchronization signal that provides a fixed global reference phase. Therefore, the data points of every time series at a given time t_j, with j in $[1, Z]$, correspond to an equivalent overall temporal phase in the oscillation of the sample. Taking slices from this cuboid, as shown in **Fig. S2(C)**, results in the images from which the displacement trajectory of the ROI can be extracted using DIC. Clearly, the number of data points Z equals the number of resulting images for each ROI. In total, one such dataset is acquired for every excitation frequency at every ROI of the sample. For the measurements presented in this work, the excitation frequency is kept constant until all ROIs have been measured for that frequency. In principle, it is also possible to go through all desired excitation frequencies before proceeding to the next ROI. However, we have made the experience that best results are

achieved when keeping the excitation frequency constant until all ROIs have been measured. Between changing the excitation frequency and starting a new time series, the data acquisition is halted for about $200\,\mathrm{ms}$. As mentioned in the main text, this waiting time acts as a buffer for the sample to respond to the new drive signal and allow for the previously excited metamaterial phonon modes to decay in time. Since the metamaterial sample exceeds the optical field of view of the setup, not all ROIs can be accessed by scanning of the laser beam directly. Therefore, the xyz-translation stage is used to position the ROI under investigation in the field of view of the setup.

Measurement parameters

To obtain the displacement field of the elastic metamaterial samples, the ROI size is chosen to span 60 by 60 pixels over a rectangular sample area of $30 \times 30\,\mu\mathrm{m}^2$. The kernel size for the DIC is chosen to span 40 by 40 pixels. A total of 51 ROIs has been measured for one experiment on one sample. The zeroth ROI is located on the base plate of the sample. This choice is useful to check the trajectory of the piezoelectric actuator before each measurement and ensure that the orientation of the sample excitation is as desired. Hence, it provides quantitative information about phase and amplitude of the sample excitation. An example of such a displacement dataset for $125\,\mathrm{kHz}$ frequency is shown in **Fig. S3**. The remaining 50 ROIs are placed on the cross shaped markers on the outer frame of each metamaterial layer. The frequency spacing of the excitation is $\Delta f = 5\,\mathrm{kHz}$. The sample rate of the DAQ was set to $10\,\mathrm{MS/s}$. For the frequency region between $50\,\mathrm{kHz}$ to $200\,\mathrm{kHz}$, $Z = 1024$ data points per pixel are acquired. To accommodate for the larger oscillation periods at excitation frequencies between $20\,\mathrm{kHz}$ and $45\,\mathrm{kHz}$, the number of data points is increased to $Z = 4200$.

White-Noise Acoustic Measurements.

An equivalent alternative to the single-frequency excitation experiments presented in the main paper is to use excitation with white acoustical noise. For the situations shown in the main paper, we have obtained equivalent results indeed. The data for higher bands in the air-borne acoustical roton metamaterial experiments have been obtained by using white-noise excitation. Here, white acoustical noise signal with a cut-off frequency of $3\,\mathrm{kHz}$ is produced by an arbitrary

frequency generator, amplified and sent to the loudspeaker. Using the sound card of the computer, the signals received by two microphones are recorded simultaneously at the Nth unit cell and at the entrance unit cell where the speaker is fixed. The cross-spectral density is estimated using Welch's method (*41*) to obtain the frequency response. This process is repeated for all of the 50 unit cells.

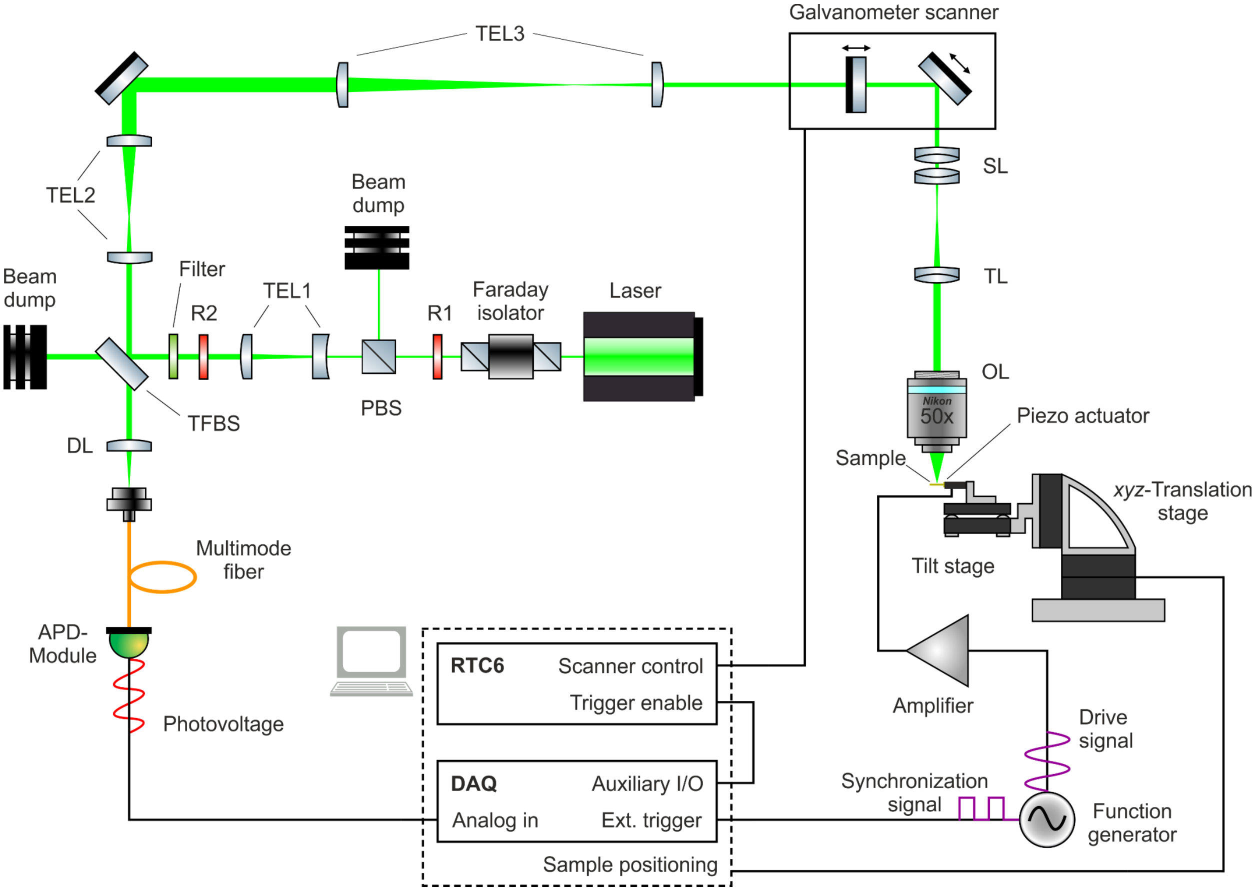

Fig. S1. Scheme of the experimental setup for investigating rotons in the microscale metamaterial samples. Beam path depicted in green. (TEL1-TEL3) telescopes for beam-size manipulation. Polarizing beam splitter cube (PBS). Half-wave retarder plates (R1, R2). Scan lens (SL), tube lens (TL), and objective lens (OL). Detection lens (DL). Components of the personal computer (PC) are shown in the dashed box: data acquisition unit (DAQ), galvanometer scanner control card (RTC6). Electrical wiring is schematically shown by the black lines.

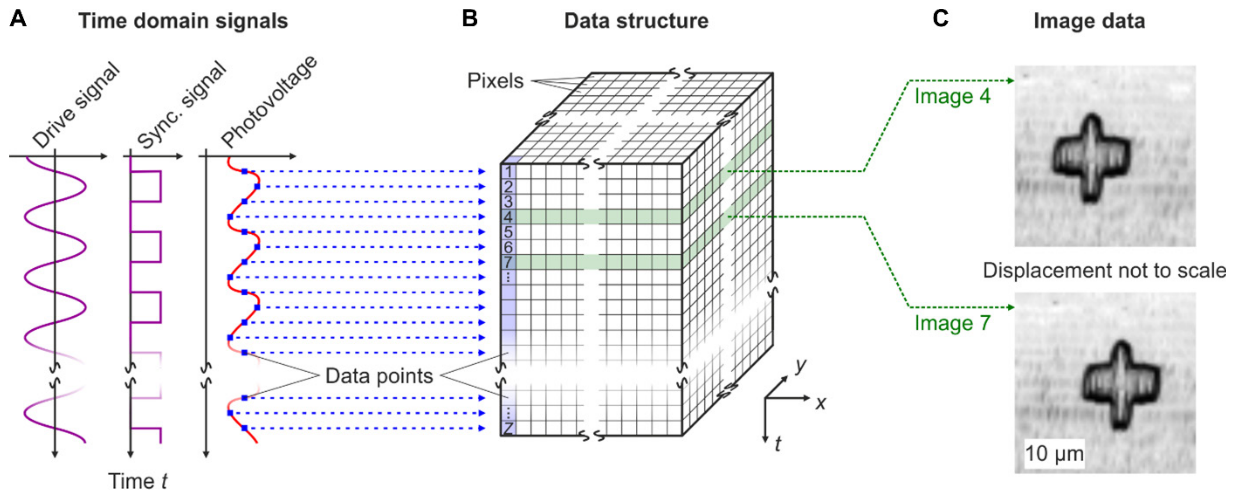

Fig. S2. Workflow from data acquisition to image generation. (**A**) Illustration of the time-domain signals required for sample excitation (drive signal), synchronization of sample excitation and data acquisition (synchronization signal), and measured signal (photovoltage) for one pixel. Data points are represented by the blue squares. (**B**) Visualization of a measurement data set for one excitation frequency and for a single range of interest. The data is structured as a cuboid. A tripod indicates the lateral $\boldsymbol{x}$- and $\boldsymbol{y}$- direction, which correspond to real-space pixel coordinates in the image plane, while the third direction represents the time axis *t*. The numbered column of cubes highlighted in blue represents a photovoltage time series of data points for a single pixel. The numbering from 1 to $\boldsymbol{Z}$ emphasizes the sequence of data acquisition per pixel. Combining the datapoints in slices of the resulting cuboid, as indicated by the two horizontally green shaded areas, produces the image data for digital image cross-correlation analysis. (**C**) Two exemplary slices are taken out of the cuboid. The shown images consist of every 4th and 6th data point taken from the individual time series respectively, as indicated by the labels. For clarity, the shown displacement is intentionally exaggerated.

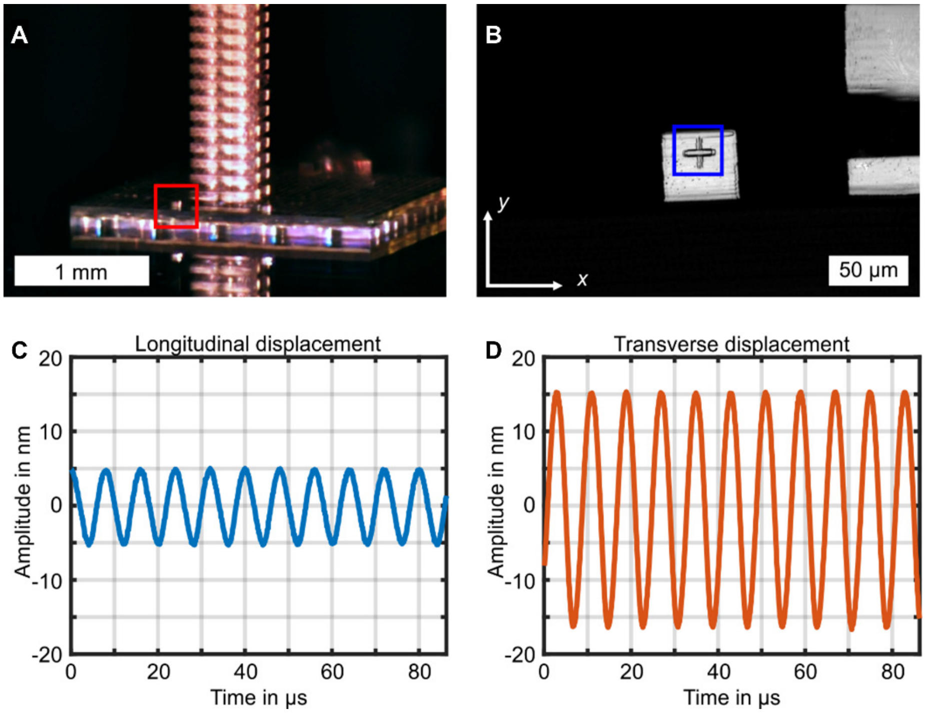

Fig. S3. Examples of displacement data. (**A**) Bright-field light-microscope image of the bottom section of a microscale metamaterial sample for the elastic-wave experiments. The red rectangle shows the region where a cross-shaped marker is located. (**B**) Image taken by the home-built confocal microscope. It shows the cross-shaped marker on a cuboid which has been printed on the bottom plate of the metamaterial sample. The bottom plate itself is not visible, since it is out of focus and hence suppressed by the axial sectioning of the confocal microscope. On the right-hand side, edges of the lowest layers of the metamaterial sample are still visible. The ROI measured for the determination of the displacement data is enclosed in the blue rectangle which is up to scale. The coordinate system defines the longitudinal $\boldsymbol{y}$-direction and the transverse $\boldsymbol{x}$-direction with respect to the sample. (**C**) Longitudinal and (**D**) transverse component of the displacement data from the excitation of the sample obtained with digital image cross-correlation analysis. A baseline subtraction using a floating average that spans one oscillation period of the excitation frequency is performed on the raw data before plotting. In this example, the excitation frequency is set to $\mathbf{125\,kHz}$. To determine the displacement amplitude and phase for the derivation of the roton band structure, a least-square fit using a sine function is performed on the data (not shown).

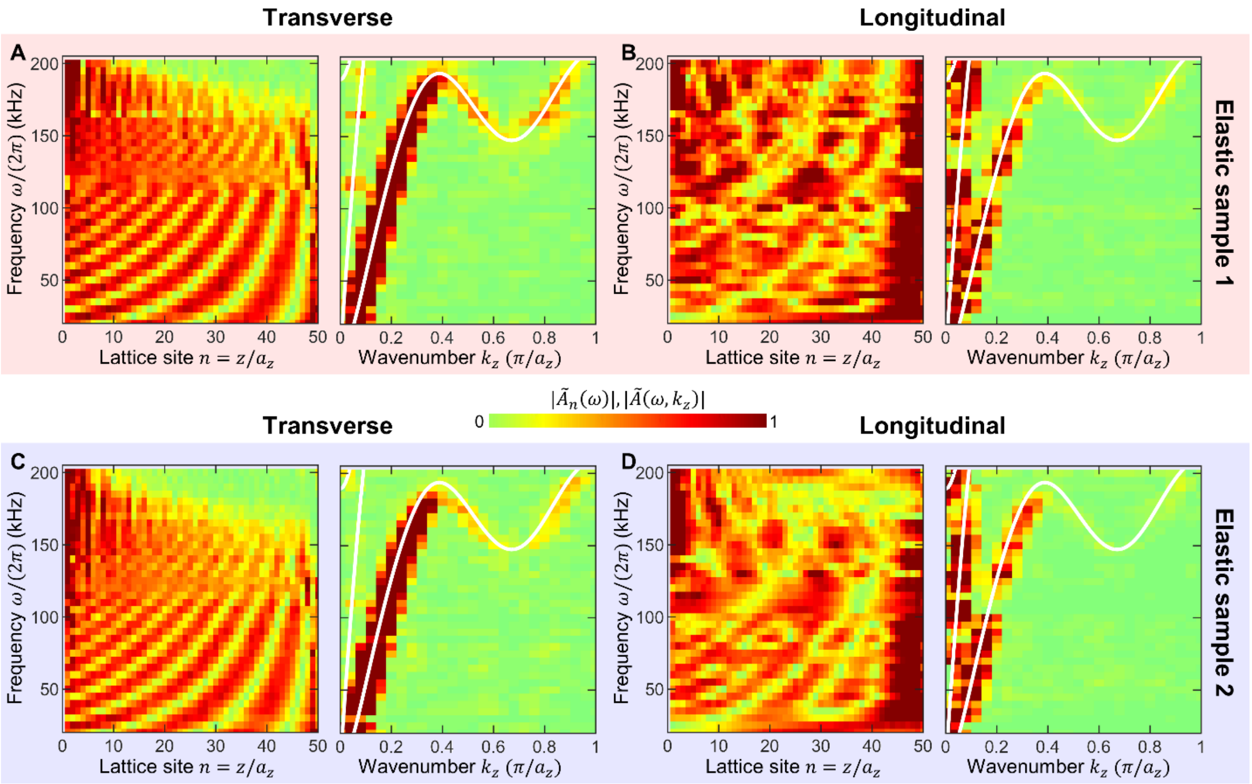

Fig. S4. Measured and calculated roton dispersions based on transverse and longitudinal displacement. (**A**) Measured transverse displacement (left) for the sample in Fig. 2 and Fig. 3 versus position and frequency and derived roton band structure (right) based on the transverse displacement. (**B**) Same as in (**A**), but for measured longitudinal displacement. (**C**), (**D**) Same as in (**A**) and (**B**), but for a different 3D printed sample.

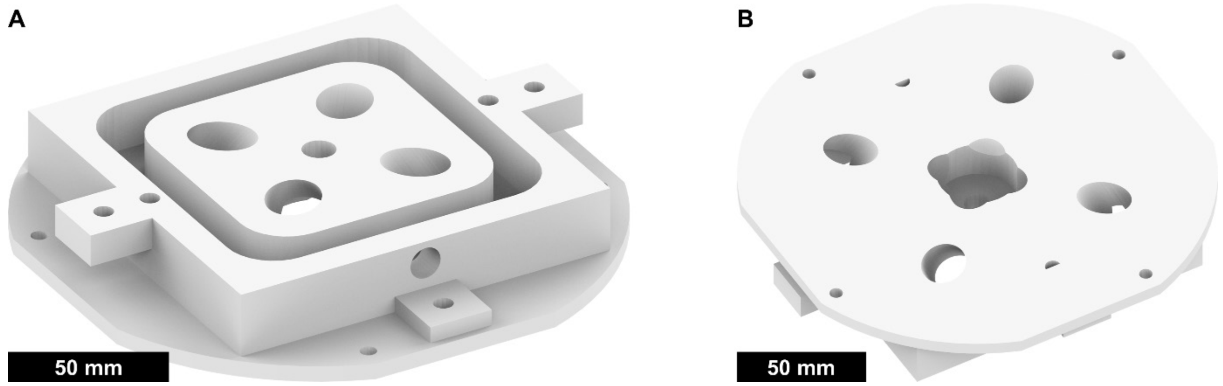

Fig. S5. Extra unit cell with a circular face to connect the loudspeaker to the airborne sound roton metamaterial. (**A**) Front-side view and (**B**) back-side view.

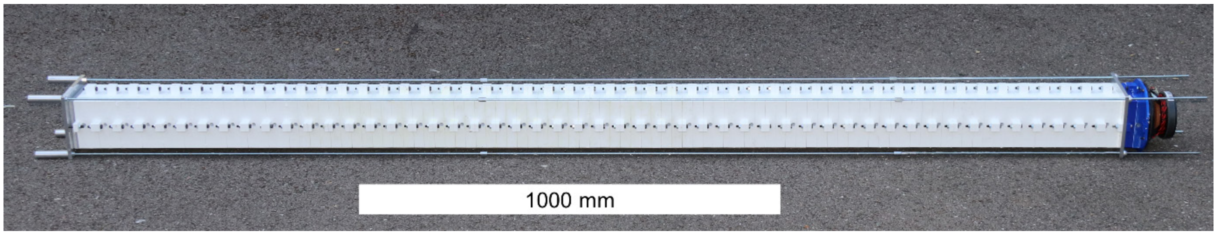

Fig. S6. Full view of the air-borne sound roton metamaterial sample.

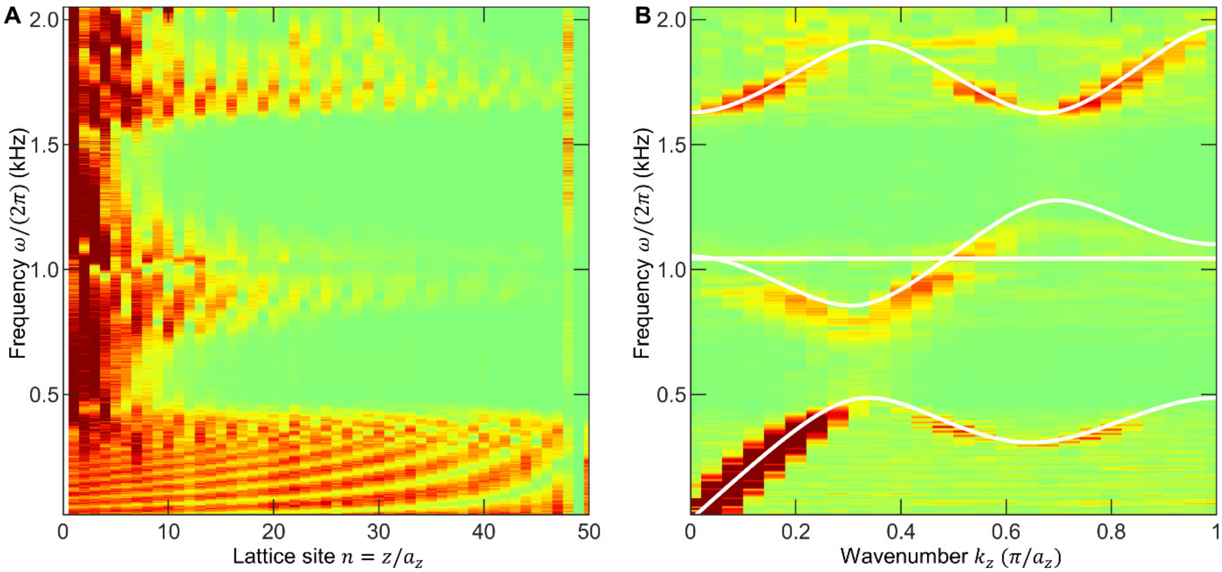

Fig. S7. Measured and calculated acoustic roton dispersions using white-noise excitation. (**A**) Measured raw data for the sample in Fig. 3 versus position and frequency. (**B**) Derived roton band structure. The solid curves are the calculated roton band structure for a lossless metamaterial beam that is infinitely extended along the $\boldsymbol{z}$-direction.

Component	Designation	Model
Laser	-	LCX-532S-200, Oxxius SA
Optical isolator	Faraday isolator	LINOS FI-530-2SV, QIOPTIQ
Halve-wave plate	R1	λ/2 532 nm low, B. Halle
Halve-wave plate	R2	WPMQ10M-532, Thorlabs
Clean-up filter	Filter	FLH532-10, Thorlabs
Polarizing beam-splitter cube	PBS	PTW15, B. Halle
Thin-film beam splitter	TFBS	BSW10, Thorlabs
Galvanometer scanner	-	intelliSCAN se 10, Scanlab
Scan lens	SC	2x AC508-250-A, Thorlabs
Tube lens	TL	AC508-200-A-ML, Thorlabs
Objective lens	OL	50X CFI60 TU Plan Epi ELWD, Nikon
Multimode fiber	-	M122L01 200μm NA 0.22, Thorlabs
Avalanche photodiode module	APD-Module	C10508-01SPL, Hamamatsu
xyz-Translation stage	-	3x Q-545.140, Physik Instrumente
Piezo actuator	-	PL055.31 PICMA®, Physik Instrumente
Signal amplifier	Amplifier	A 1230-01 linear amplifier, Hubert
Function generator	-	33612A, Keysight
Data acquisition unit	DAQ	ATS9130, AlazarTech
Scanner control card	RTC6	RTC6, Scanlab

Table S1. List of components used in the scanning-confocal-optical-microscope setup. The table provides the model and manufacturer information of key components used in the home-built confocal-laser-scanning-microscopy setup, along with the component abbreviations used in the text.

REFERENCES AND NOTES

1. L. Landau, Theory of the superfluidity of helium II. *Phys. Rev.* **60**, 356–358 (1941).

2. R. P. Feynman, Atomic theory of the two-fluid model of liquid helium. *Phys. Rev.* **94**, 262–277 (1954).

3. R. P. Feynman, Atomic theory of liquid helium near absolute zero. *Phys. Rev.* **91**, 1301–1308 (1953).

4. D. G. Henshaw, A. D. B. Woods, Modes of atomic motions in liquid helium by inelastic scattering of neutrons. *Phys. Rev. Lett.* **121**, 1266–1274 (1961).

5. A. Griffin, G. Allan, *Excitations in a Bose-Condensed Liquid* (Cambridge Univ. Press, 1993).

6. N. Bogoliubov, On the theory of superfluidity. *J. Phys.* **11**, 23 (1947).

7. R. P. Feynman, M. Cohen, Energy spectrum of the excitations in liquid helium. *Phys. Rev.* **102**, 1189–1204 (1956).

8. T. Schneider, C. P. Enz, Theory of the superfluid-solid transition of ^{4}He. *Phys. Rev. Lett.* **27**, 1186–1188 (1971).

9. H. R. Glyde, A. Griffin, Zero sound and atomiclike excitations: The nature of phonons and rotons in liquid ^{4}He. *Phys. Rev. Lett.* **65**, 1454–1457 (1990).

10. G. J. Kalman, P. Hartmann, K. I. Golden, A. Filinov, Z. Donkó, Correlational origin of the roton minimum. *Europhys. Lett.* **90**, 55002 (2010).

11. A. D. B. Woods, Neutron inelastic scattering from liquid helium at small momentum transfers. *Phys. Rev. Lett.* **14**, 355–356 (1965).

12. K. Beauvois, J. Dawidowski, B. Fåk, H. Godfrin, E. Krotscheck, J. Ollivier, A. Sultan Microscopic dynamics of superfluid ^{4}He: A comprehensive study by inelastic neutron scattering. *Phys. Rev. B* **97**, 184520 (2018).

13. H. J. Maris, Phonon-phonon interactions in liquid helium. *Rev. Mod. Phys.* **49**, 341–359 (1977).

14. H. Godfrin, K. Beauvois, A. Sultan, E. Krotscheck, J. Dawidowski, B. Fåk, J. Ollivier, Dispersion relation of Landau elementary excitations and thermodynamic properties of superfluid He 4. *Phys. Rev. B* **103**, 104516 (2021).

15. H. Godfrin, M. Meschke, H. J. Lauter, A. Sultan, H. M. Böhm, E. Krotscheck, M. Panholzer, Observation of a roton collective mode in a two-dimensional Fermi liquid. *Nature* **483**, 576–579 (2012).

16. T. R. Koehler, N. R. Werthamer, Computation of phonon spectral functions and ground-state energy of solid Helium. I. bcc phase. *Phys. Rev. A* **5**, 2230–2237 (1972).

17. S. M. Girvin, A. H. MacDonald, P. M. Platzman, Magneto-roton theory of collective excitations in the fractional quantum Hall effect. *Phys. Rev. B* **33**, 2481–2494 (1986).

18. I. V. Kukushkin, J. H. Smet, V. W. Scarola, V. Umansky, K. von Klitzing, Dispersion of the excitations of fractional quantum Hall states. *Science* **324**, 1044–1047 (2009).

19. I. V. Kukushkin, V. Umansky, K. von Klitzing, J. H. Smet, Collective modes and the periodicity of quantum Hall stripes. *Phys. Rev. Lett.* **106**, 206804 (2011).

20. N. Henkel, R. Nath, T. Pohl, Three-dimensional roton excitations and supersolid formation in Rydberg-excited Bose-Einstein condensates. *Phys. Rev. Lett.* **104**, 195302 (2010).

21. M. Lahrz, M. Lemeshko, L. Mathey, Exotic roton excitations in quadrupolar Bose–Einstein condensates. *New J. Phys.* **17**, 45005 (2015).

22. L.-C. Ha, L. W. Clark, C. V. Parker, B. M. Anderson, C. Chin, Roton-maxon excitation spectrum of Bose condensates in a shaken optical lattice. *Phys. Rev. Lett.* **114**, 55301 (2015).

23. L. Chomaz, R. M. W. van Bijnen, D. Petter, G. Faraoni, S. Baier, J. H. Becher, M. J. Mark, F. Wächtler, L. Santos, F. Ferlaino, Observation of roton mode population in a dipolar quantum gas. *Nat. Phys.* **14**, 442–446 (2018).

24. D. Petter, G. Natale, R. M. W. van Bijnen, A. Patscheider, M. J. Mark, L. Chomaz, F. Ferlaino, Probing the roton excitation spectrum of a stable dipolar Bose gas. *Phys. Rev. Lett.* **122**, 183401 (2019).

25. J. N. Schmidt, J. Hertkorn, M. Guo, F. Böttcher, M. Schmidt, K. S. H. Ng, S. D. Graham, T. Langen, M. Zwierlein, T. Pfau, Roton excitations in an oblate dipolar quantum gas. *Phys. Rev. Lett.* **126**, 193002 (2021).

26. J. Kishine, A. S. Ovchinnikov, A. A. Tereshchenko, Chirality-induced phonon dispersion in a noncentrosymmetric micropolar crystal. *Phys. Rev. Lett.* **125**, 245302 (2020).

27. Y. Chen, M. Kadic, M. Wegener, Roton-like acoustical dispersion relations in 3D metamaterials. *Nat. Commun.* **12**, 3278 (2021).

28. R. Fleury, Non-local oddities. *Nat. Phys.* **17**, 766–767 (2021).

29. S. A. Cummer, J. Christensen, A. Alù, Controlling sound with acoustic metamaterials. *Nat. Rev. Mater.* **1**, 16001 (2016).

30. K. Bertoldi, V. Vitelli, J. Christensen, M. Van Hecke, Flexible mechanical metamaterials. *Nat. Rev. Mater.* **2**, 17066 (2017).

31. A. C. Eringen, Linear theory of micropolar elasticity. *J. Math. Mech.* **15**, 909–923 (1966).

32. A. C. Eringen, *Microcontinuum Field Theories* (Springer, 1999).

33. Y. Chen, T. Frenzel, S. Guenneau, M. Kadic, M. Wegener, Mapping acoustical activity in 3D chiral mechanical metamaterials onto micropolar continuum elasticity. *J. Mech. Phys. Solids* **137**, 103877 (2020).

34. J. O. Smith III, *Spectral Audio Signal Processing* (W3K publishing, 2011).

35. T. Frenzel, J. Köpfler, A. Naber, M. Wegener, Atomic scale displacements detected by optical image cross-correlation analysis and 3D printed marker arrays. *Sci. Rep.* **11**, 2304 (2021).

36. B. Pan, K. Qian, H. Xie, A. Asundi, Two-dimensional digital image correlation for in-plane displacement and strain measurement: A review. *Meas. Sci. Technol.* **20**, 062001 (2009).

37. C. Jordan, K. Jordán, *Calculus of Finite Differences* (American Mathematical Soc., 1965).

38. C. E. Shannon, Communication in the presence of noise. *Proc. IRE* **37**, 10–21 (1949).

39. T. Frenzel, J. Köpfler, E. Jung, M. Kadic, M. Wegener, Ultrasound experiments on acoustical activity in chiral mechanical metamaterials. *Nat. Commun.* **10**, 3384 (2019).

40. A. Negrean, H. D. Mansvelder, Optimal lens design and use in laser-scanning microscopy. *Biomed. Opt. Express* **5**, 1588–1609 (2014).

41. P. Welch, The use of fast Fourier transform for the estimation of power spectra: A method based on time averaging over short, modified periodograms. *IEEE Trans. Audio Electroacoust.* **15**, 70–73 (1967).
